# The Role of Extracellular Heat Shock Proteins in Cardiovascular Diseases

**DOI:** 10.3390/biomedicines11061557

**Published:** 2023-05-27

**Authors:** Soumya Patnaik, Sriram Nathan, Biswajit Kar, Igor D. Gregoric, Yi-Ping Li

**Affiliations:** 1 Division of Cardiology, The University of Texas Health Science Center at Houston, Houston, TX 77030, USA; Soumya.Patnaik@uth.tmc.edu; 2 Department of Advanced Cardiopulmonary Therapies and Transplantation, The University of Texas Health Science Center at Houston, Houston, TX 77030, USA; 3 Division of Integrative Biology and Pharmacology, The University of Texas Health Science Center at Houston, Houston, TX 77030, USA

**Keywords:** heat shock proteins, apoptosis, atherosclerosis, heart failure, ischemia-reperfusion injury, immune cell activation, stress

## Abstract

In the early 1960s, heat shock proteins (HSPs) were first identified as vital intracellular proteinaceous components that help in stress physiology and reprogram the cellular responses to enable the organism’s survival. By the early 1990s, HSPs were detected in extracellular spaces and found to activate gamma-delta T-lymphocytes. Subsequent investigations identified their association with varied disease conditions, including autoimmune disorders, diabetes, cancer, hepatic, pancreatic, and renal disorders, and cachexia. In cardiology, extracellular HSPs play a definite, but still unclear, role in atherosclerosis, acute coronary syndromes, and heart failure. The possibility of HSP-targeted novel molecular therapeutics has generated much interest and hope in recent years. In this review, we discuss the role of Extracellular Heat Shock Proteins (Ec-HSPs) in various disease states, with a particular focus on cardiovascular diseases.

## 1. Introduction

Heat shock proteins (HSPs) were first described in 1962 as temperature-sensitive proteins in *Drosophila*. Later, similar molecules were observed in many organisms, including humans [1]. In humans, HSPs were mainly studied as intracellular components, helping other proteins maintain their structure and function under stress conditions, including physiological stress, mechanical stress, environmental stress (heat, cold, ultraviolet light), and infections [2]. In 1991, extracellular HSPs were found to have an immune-regulatory role, and they have been linked to several disease entities since that time [3]. 

The contribution of extracellular HSPs to health and disease is not yet fully understood and requires further investigation. This review analyses this fast-expanding area of research and discusses the current perspectives and gaps in knowledge about the function of extracellular HSPs in various disease states and in cardiovascular diseases in particular.

## 2. Biochemistry and Types of HSPs and Related Molecules

When various insults challenge cells, they produce a family of polypeptides identified as stress proteins, or HSPs. The generation of HSPs is a basic and well-conserved cellular response seen in plants, animals, and human beings. It is well known that HSPs in the intracellular space have a vital role in cell repair following an injury and help prevent future insults (stress tolerance) [4].

The predominant intracellular function of HSPs is to act as “molecular chaperones” in the cytosol. Research has shown that HSPs aid the cell in folding polypeptides, repair/refold/degrade misfolded proteins, and regulate apoptosis [4,5,6]. Lower concentrations of HSPs are found in the cells during the physiological conditions involved in cellular processes like protein folding, the assembly of macromolecule complexes, and signal transduction [5].

A consensus nomenclature was proposed in 2009 that grouped HSPs into discrete families based on their molecular mass [7] (Table 1). There are several isoforms of HSPs, but only a few specific isoforms are involved in stress physiology. 

### 2.1. Extracellular Heat Shock Proteins [Ec-HSPs]

HSPs were thought to be exclusive to the intracellular space until HighTower and Guidon detected HSP70 (HSPAI) in an extracellular medium in 1989 [8]. This initial finding was regarded as an artifact and not given much attention for several years. However, by 2000, interest in extracelluar HSPs had grown as additional evidence became available. First, the release of HSP70 during tissue necrosis and activation of macrophages was documented [9]. Furthermore, HSP60 (HSPD 1), normally an intra-mitochondrial protein, was detected in extracellular spaces in some disease conditions, and circulating anti-HSP 60 antibodies were detected in the blood [10,11,12]. Three mechanisms have been described to enable the release of HSPs from the intracellular to the extracellular space: (a) translocation across the plasma membranes; (b) release associated with lipid vesicles; and (c) passive release after cell death by necrosis.

**Figure 1:**
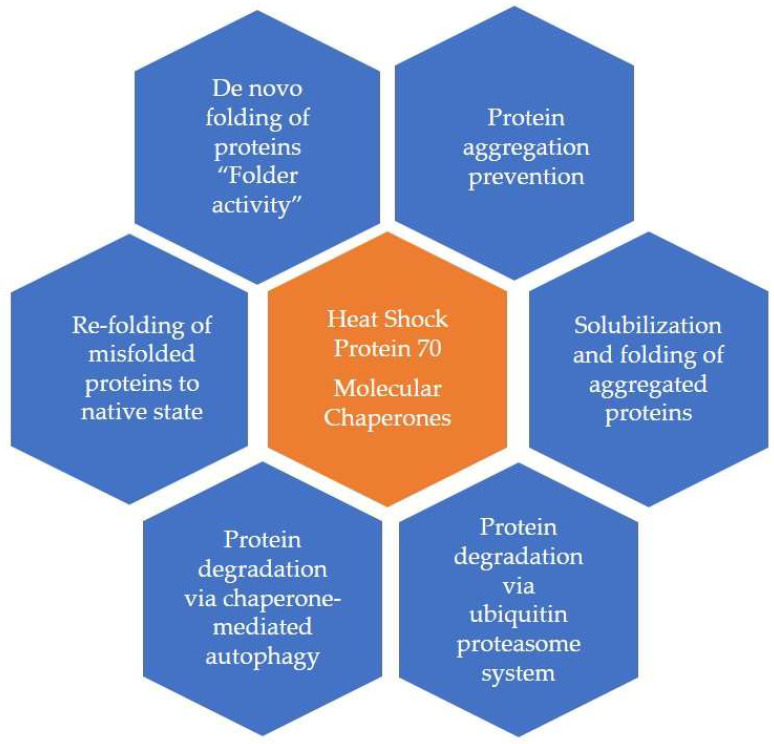
Molecular Mechanisms of Heat Shock Protein 70.

HSP70 and HSP60 have been the most detected and described HSPs in the literature [8,13]. They act as alert stress signals that prime other cells, particularly the immune system. The molecular mechanisms of HSP70 are depicted in Figure 1. Ec-HSPs (HSP70, HSP60, and Grp 96) play a role that is similar to that of the danger-associated molecular pattern (DAMP) protein and modulate the immune response [14]. The target cells express certain receptors like LOX-1, FEEL-1, SRA-1, and SREC-1 that can bind HSPs; these receptors can be sorted into two classes: (1) C-type lectin receptors (CLR) and (2) scavenger receptors (SR) [13]. HSP70 and 90 activate toll-like receptors (TLRs), including TLR2 and TLR4. HSP-chaperoned molecules also play a role in innate immune responses and antigen cross-presentation within antigen-presenting cells [15].

### 2.2. Other HSP70-like Molecules

Various other intracellular proteins like Grp78 (HSPA5) and Grp75 (Mortalin), which are homologous to HSP70, were also detected in extracellular locations. HSP90 was first detected on the surface of tumor cells and subsequently in multipotential mesenchymal precursor cells, human neuroblastoma cells, and human monocytes; it is considered a product of cell death, but its exact role is not understood [16]. 

### 2.3. Co-Chaperones

Co-chaperones are proteins that are non-client-binding partners of the chaperones, HSP70 or HSP90. They may interact with HSP70, HSP90, or both. Recently, the carboxy-terminus of the HSP70-interacting protein (CHIP) and BCL2-associated athanogene 3 (BAG-3) were recognized for their role in cardiomyocyte integrity during stress [17]. Other co-chaperones include Hsp40, GrapE, BAG1, and BAG2.

### 2.4. Small HSPs 

HSPs with a molecular weight of 15 to 49 kDa are small HSPs (sHSP, also called HSP family B) and have emerged as critical regulators of protein folding. They have either BAG3-dependent (HSPB5,6,8) or independent (HSBP7) actions that regulate the cardiac stress response. HSP27 (HSPB1) was detected in the patient’s serum in some diseases, such as chronic pancreatitis and pancreatic carcinoma [18].

In summary, HSPs appear to be a complex group of molecules that take part in stress physiology and help reprogram cellular responses to enable the organism’s survival.

## 3. Role in Non-Cardiac Disorders

Under normal physiology, the production of HSPs accounts for less than 10% of the total protein content; during times of stress, this value can increase to 15% or more [19]. 

The number of clinical conditions associated with Ec-HSPs is growing, and the list includes cancer, diabetes, chronic inflammatory and immune disorders, trauma, and diseases that affect the cardiovascular, renal, hepatic, and pulmonary systems (Table 2) [16,20,21,22]. 

A raised level of Hsp70 in the plasma has been noted in pregnancy, following heavy exercise, and in acute infections. In critically ill patients, a raised Hsp70 level correlates with improved survival [23]. Several studies have demonstrated that Hsp70 has an immune regulatory role [24]. Further, Hsp70 has been shown to induce the activation of macrophages, monocytes, dendritic cells, and natural killer cells. Increased microbial capacity, neutrophilic chemotaxis, phagocytosis, monocyte response to endotoxins, and immunomodulatory activities were all associated with increased levels of extracellular Hsp70 [16,25]. Cancer cells express high levels of Hsp70 at different stages of tumor formation and during therapy [26]; thus, Hsp70 has also been associated with the growth of tumor cells and apoptosis resistance [27]. Hsp70 is reported to activate cancer cell motility, migration, and metastasis. HSPs also inhibit apoptosis and promote antioxidant defence [28]. Likewise, increased levels of Hsp27 have been documented in tumors of the prostate, breast, uterus, ovary, head and neck, gastrointestinal tract, Hodgkin’s disease, and various tumors of the CNS and bladder [29,30].

Human Epidermal Growth Factor Receptor-2 (HER2) plays an important role in stages of cell development by influencing downstream signal proteins; its mutation or overexpression leads to tumorigenesis and metastasis. HER2 activity itself is associated with Hsp90 [31]. Tumor-released extracellular Hsp70 and Hsp90 stimulated catabolism, resulting in muscle wasting and causing systemic inflammation [32]. Elevated circulating Hsp70 and 90 levels have been found to increase with the pathological grade and stage of cancer [33,34,35], and they are associated with cancer-induced muscle wasting in mice [32,36,37]. Increased levels are also observed in cancer patients with weight loss [38]. Elevated serum levels of Hsp90 have been detected in patients with rheumatoid arthritis, and they have been shown to activate macrophages [39]. Elevated levels of Hsp90 were detected in the serum of patients with systemic lupus erythematosus [40]. Hsp60 was detected in the saliva and serum of type 2 DM, but Hsp70 was detected in diabetic ketoacidosis [41,42]. Hsp70 can enhance alcohol- or *H. pylori*-associated gastritis and, on the contrary, may strengthen the gastric defence system [43]. Raised Hsp27 is reported in inflammatory bowel disorders, hepatic dysfunction, and pancreatitis [18].

In some cellular models of spinal and bulbar muscular atrophy, a type of motor neuron disease, Hsp70 and Hsp40 were overexpressed; they inhibited the accumulation of abnormal polyglutamine proteins and thus inhibited cell death [44].

In Alzheimer’s disease and Parkinson’s disease, the two important neuro-degenerative disorders, recent research confirmed the protective role of HSPs, which influence the folding of proteins and deliver misfolded proteins to the ubiquitin-proteasome system for degradation [45].

## 4. Ec-HSPs and Cardiovascular Diseases

It is increasingly recognized that cellular stresses lead to an accumulation of misfolded proteins during the development of cardiac hypertrophy, heart failure, and ischemia-reperfusion injury. As early as 1988, Currie et al. reported that elevated Hsp70 levels are associated with better recovery from ischemic insult in rats [46]. The initial compensatory response is increased chaperones/co-chaperones, which reverse/reduce these processes to some extent. When the compensatory mechanisms become overwhelmed, cardiac dysfunction can occur [47]. Growing evidence shows that cardiac protection can be enhanced by regulating the activity of chaperones and their related substances. Only in the past few years have the major HSPs (Hsp70 and Hsp90) and co-chaperones (CHIP and BAG-3) been shown to play a crucial role in maintaining cardiac integrity during stress [48]. Their role in cardiac ischemia and heart failure is also slowly unfolding, although the precise mechanism remains unclear [48]. 

**Figure 2:**
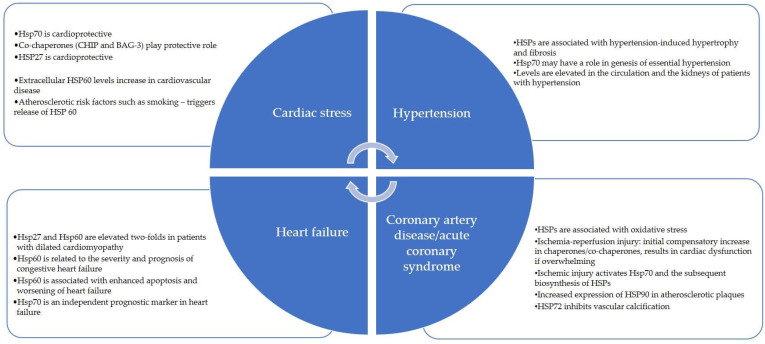
Heat Shock Proteins in Cardiovascular Diseases.

HSP90 and HSP60 can facilitate angiotensin-2 activity, resulting in myocardial injury and endothelial dysfunction in the cardiovascular system [49]. HSP70 is protective against angiotensin-2-mediated vascular smooth muscle hypertrophy and hypertension [50]. Important Ec-HSPs that have a role in cardiovascular disorders have been summarized in Table 2 and Figure 2.

**Table 2:** Classification of important extracellular heat shock proteins that have a role in cardiovascular disorders.

Family	Important Members	Major Role	Reference
Hsp90	Hsp90α (HSPC2)	Role in cytoprotection, vascular relaxation, atherosclerosis, and systemic lupus erythematosus.	[17,39,51]
	Hsp90β (HSPC3)		
	Grp94 (HSPC4)		
Hsp70 and HSP70- like molecules	Hsp70 (Hsp72) (HSPA1)	Role in atherosclerosis, heart failure, and hypertension; possible auto-antigen	[17,50,52,53,54,55,56,57]
	Hsp70 (Hsp73) (HSPA8)		
	Grp78 (BIP) (HSPA5)		
	Utp (Grp75) (HSPA9)		
Hsp60	Hsp60—mostly intracellular	Released during cell necrosis; role in atherosclerosis, heart failure, rheumatoid arthritis, diabetes mellitus, and central nervous system disorders.	[10,11,58,59,60,61]
Hsp40	Hsp40 (Dnaj) (DNAJB1)	Collagen preservation	[62]
Small Hsp	αCrystallin (HSPB4)	Hsp27 has a protective role in atherosclerosis; antioxidant functions; elevated in various cancers; inhibits apoptotic pathways. Hsp22 has recently been shown to play an important role in cardiomyopathies and age-related cardiac affections.	[63,64]
	Hsp25 (HSPB1)		
	Hsp27 (HSPB2)		
	Hsp20 (HSPB6)		
	Hsp22 (HSPB8)		
Co-chaperones	CHIP, BAG 3, Hsp40, GrapE, BAG1, BAG2	Interact with HSP70 and HSP90 during stress.	[17]

## 5. Heart Failure

Like in Alzheimer’s disease and other neurodegenerative conditions, targeting the protein quality control system in heart failure is a novel therapeutic approach. This is supported by several important observations made on HSPs and their effects on heart failure.

Misfolded proteins can be produced by myocardial infarction, heart failure, genetic mutations, or aging [65]. The presence of misfolded proteins is toxic to cardiomyocytes (direct effect) and can result in proteinopathy and heart failure [47,58]. Ec-HSP can affect apoptosis as well as cardiomyocyte contractile function in patients with heart failure [52]. In a study by Knowlton et al., the expression of various HSPs was compared among patients with dilated cardiomyopathy (DCM), those with ischemic cardiomyopathy, and control samples [53]. Hsp72, Hsp70, and Hsp90 were not significantly different in the three groups. Hsp27 and Hsp60 were elevated two-fold in DCM patients compared to control subjects [53]. In another study of 112 congestive heart failure patients, Niizeki T et al. observed that Hsp60 was related to the severity and prognosis of congestive heart failure and associated with a high risk of advanced heart failure [54]. The source of circulating Hsp70 in congestive heart failure is controversial; but believed to be produced by white blood cells via activation of the CD14 receptor in the myocardium or endocardium [55,66].

## 6. HSP70

Levels of HSP70 are elevated in chronic heart failure patients with cachexia. Some heart failure studies support using Hsp70 as a potential marker for disease severity; however, levels do not predict survival [59]. In contrast, a study by Jenei et al. showed that HSP70 is an independent predictor of mortality [67]. In another study of 222 patients, the screening value of Hsp70 was evaluated at different stages of heart failure [60]. These authors observed that Hsp70 was positively correlated with the severity of heart failure and N-terminal pro-brain natriuretic peptide (NT-proBNP) levels. However, Hsp27 and Hsp90 did not show such a correlation. They suggested that Hsp70 is a potential screening biomarker for the early diagnosis of heart failure [60]. 

Hsp70 can be induced by ischemia, nutrient deprivation, irradiation, infections, and inflammations. It is believed that its upregulation helps cell survival in the face of stressful insults. A homologue of Hsp70, heat shock cognate (HSC) 70, is constitutively present in the heart. Along with stress-induced Hsp70, HSC 70 helps in cardioprotective actions, and their action is coordinated by the other co-chaperones, Hip and Hop.

## 7. HSP60

Hsp60 plays a role in the initiation and progression of heart failure and atherosclerosis; it is found to be translocated to the myocardial surface before its release into circulation in patients with heart failure [68]. Many recognized atherosclerotic risk factors, such as smoking, chlamydial infections, and shear stress, can promote the expression of Hsp60 [69]. Increased levels of Hsp60 were shown to be associated with enhanced apoptosis and worsening of heart failure [70]. Bonanad et al. found that Hsp60 was correlated with a higher risk of subsequent death or hospital readmission. High levels of Hsp60 were associated with high troponin I levels, a low relative lymphocyte count, and clinical signs of congestion [71]. 

## 8. HSP90

Recent studies have also focused on the many cardiomyopathies and vascular remodeling pathways. They suggest a central role for HSP90 in the pathogenesis of cardiomyopathy of multiple etiologies and pulmonary arterial hypertension (PAH) [56,72]. In arterial hypertension, elevated levels of HSP90α have been detected. This is thought to be a compensatory mechanism for reduced nitric oxide bioavailability [73]. Therefore, HSP90α may be an early marker of endothelial injury in hypertensive individuals. Studies indicate that HSP90 has a role in AngII-induced vascular smooth muscle cell (VSMC) proliferation and remodeling [74,75,76]. In patients with PAH, HSP90 levels are elevated in the walls of the plasma membrane as well as the pulmonary arteriolar walls [77]. HSP 90 may also affect the metabolism of low-density cholesterol and play a role in atherosclerosis [78].

## 9. HSP 22 

The role of HSP22 is becoming clearer in recent studies, and it appears to be involved in several functions such as modulating gene transcription, post-translational modification, protein degradation, mitochondrial function, autophagy, ROS production, and anti-apoptic activities. The deletion of HSP22 has been associated with acceleration of dilated cardiomyopathy, cardiac hypertrophy, ischemic heart disease, age-related cardiomyopathy, and diabetic cardiomyopathy, and it is proven that HSP22 is protective for stressed hearts.

## 10. Cachexia

Von Haehling et al. observed that 10% of heart failure patients present with cachexia, which is attributed to multiple factors like systemic inflammatory activity, autonomic dysfunction, up-regulation of RAS, dysregulation of the immune system, and anabolic and catabolic imbalance [61]. However, the exact relationship between HSPs and chronic heart failure-induced cachexia is not well understood.

## 11. Atherosclerosis and Coronary Artery Disease

Several stressors (i.e., hypercholesterolemia, local injury, tobacco use/smoking, and toxins) result in arterial wall remodelling and over-expression of HSPs, which are processed by macrophages and presented to T and B lymphocytes [79]. HSPs activate the innate immune system to produce cytokines in patients with coronary artery disease [80]. The production of cytokines such as TNF-alpha, IL-12, and IL-15 is an important component of atherosclerosis. Specifically, Hsp60 selectively accumulates in atheromatous lesions, suggesting it stimulates atherogenic action. In early lesions, only dendritic cells express Hsp70. In advanced atherosclerosis stages, Hsp70 is abundant in dendritic cells, but it is also expressed in monocytes, macrophages, and smooth muscle cells [57]. Associated anti-Hsp antibodies correlate with the severity of atherosclerosis [81], and this has been validated in a study of 750 patients [82]. Thus, anti-Hsp antibodies could be used to screen at-risk patients for atherosclerosis. Birnie et al. have shown that anti-Hsp65 antibodies also correlate with the severity of atherosclerosis [83].

## 12. Acute Coronary Syndromes 

Increased reactive oxygen species and the development of oxidative stress are of paramount importance in the pathophysiology of ischemia-reperfusion injury. Publications highlight an association between HSPs and oxidative stress [84]. Overall, the theory is that HSPs are cardioprotective and help in recovery after injury. Hsp70 expression is elevated in the myocardium following coronary artery bypass surgery, any aortic cross-clamping surgery, or in the setting of ischemia. Ischemic preconditioning and exercise also release Hsp70. Interestingly, patients with higher levels of Hsp70 in the circulation developed less post-operative atrial fibrillation [85]. 

When reperfusion is established after 20 min of ischemia, there is a several-fold surge of Hsp70 and Hsp90 mRNA. A concurrent increase in a transcription factor called heat shock factor 1 (HSF1) that is released due to the accumulation of reactive oxygen species during ischemia-reperfusion injury is thought to be related [86]. In animal studies, Hsp70 and Hsp72 were proven to have cardioprotective effects in ischemia-reperfusion injuries by their close interaction with antioxidant and nitric oxide generation [87]. It is believed that the deficiency of antioxidants that occurs during an ischemia/reperfusion injury activates Hsp70 and the subsequent biosynthesis of these HSPs [87]. The cardioprotective effects of Hsp70 and 90 in ischemia-reperfusion injury are manifested as increased cell resistance to hypoxia and oxidative stress and an increase in functional recovery with infarction size [88].

Animal models have shown that necrotic tissue releases Hsp70 [13]. It has also been observed that in acute myocardial infarction, there is a significant extracellular Hsp70 due to additional active secretory mechanisms besides necrosis.

## 13. Hypertension

Hsp70 is elevated in the circulation and kidneys of patients with hypertension [89]. In animals, HSPs can induce renal inflammation and hypertension [63]. Specific peptide sequences of Hsp70 prevent and correct salt-induced hypertension [63]. Autoimmune reactivity against Hsp70 may play a role in the genesis of essential hypertension. Genetic polymorphisms of Hsp70 are associated with essential hypertension [89]. Ec-HSP is also associated with hypertension-induced hypertrophy and fibrosis [63,90]. It is well known that the magnitude and rapidity of Hsp70 elevation determine the impact on the properties of the vascular smooth muscle. Potential therapeutic uses of Hsp70 in essential hypertension deserve to be investigated [91]. In addition, HSP90 is now found to be associated with the endothelial dysfunction involved in systemic hypertension; expression in this situation may be a compensatory mechanism [73].

## 14. Chronic Atrial Fibrillation

When the atrial cardiomyocytes are stressed, there is proteostasis derailment and remodelling, leading to persistent atrial fibrillation. In the initial stages of atrial fibrillation, HSP27 is elevated as a protective phenomenon; when the persistent stage is reached, its levels are exhausted [51]. Increased expression of Hsp27 protects the atrial myocytes from myolysis and may therefore slow the progression to persistent atrial fibrillation [51]. Studies have reported an upregulation of the expression of Hsp60, Hsp10, and Hsp75/mortalin in those with chronic atrial fibrillation [92,93]. Some studies indicate that alteration in the expression of Hsp60 is associated with various degrees of atrial myolysis in different stages of atrial fibrillation [94]. 

## 15. Emerging Diagnostic/Therapeutic Possibilities

With new evidence on the role of various HSPs in disease conditions, novel diagnostic and therapeutic possibilities are surfacing. The elevation of HSPs in plasma can be used as biomarkers for heart failure and atrial fibrillation [48]. Ec-HSP70 and BAG3 are identified as independent prognostic markers of mortality with heart failure and cardiac arrest [95]. BAG-3 has a role in nitric oxide release and is identified in advanced heart failure. It can be useful as a biomarker for advanced heart failure, and has the potential to be used as a novel anti-hypertensive agent [96].

HSPs can potentially be therapeutically modulated in heart failure patients to fix the imbalance of protein damage and endogenous protein quality control systems [48]. Promising in vitro data have recently identified future avenues for HSP-driven therapeutics. Since Hsp70 protects tissue from ischemic reperfusion injuries, interventions to increase certain HSPs are more likely to succeed in acute ischemic syndromes than in chronic disease states. Ec-HSPs can confer cardiac protection against various insults to the myocardium. Doxorubicin-induced cardiotoxicity was reduced by extracellular Hsp25 in a murine study [97]. Geranyl-geranyl-acetone (GGA) was recently proposed as a potential cardioprotective agent due to its property of inducing HSF1, Hsp72, and Hsp70 mRNA [98]. In animals with DCM or pressure-overloaded ventricles, Hsp70 worsens heart failure [62,99]; thus, overexpression may exacerbate chronic conditions. It is also found that Hsp-inhibitors (17-AA and 17-DMAG) reduce inflammatory responses in atherosclerosis [100]. Transcriptional inhibition of intracellular Hsp70 reduced myocyte fibrosis, but inhibiting Ec-HSP with anti-Hsp70 antibodies attenuated hypertension-induced cardiac hypertrophy and fibrosis [90]. Inhibition of extracellular Hsp90 was noted to decrease collagen production and reduce fibrosis. Experiments were conducted on several HSP90 inhibitors, including Geldanamycin, 17-AGG, 17-DMAG, Gamitrinib, and Celastrol. Although initial preclinical observations were promising, Hsp90 inhibitors are still in the experimental stage due to the fear of unknown side effects. These proteins could be a potential target in the future [101,102].

Statins, the backbone of dyslipidemia management, have activities beyond decreasing cholesterol, such as immune modulation, reduction of apoptosis, and nitric oxide production. Statins also induce HSPs (Hsp70, Hsp90, and sHsps), but the implication of this effect of statins on the therapy of cardiac disease has not yet been identified [103]. In a study by Forouzanfar et al., upregulation of endothelial thermomodulin was demonstrated with the use of statins by activating specific heat shock elements, nitric oxide-dependent dissociation of HSF1 from HSP90, and nuclear translocation of HSF1 [104]. Statins were also associated with a reduction in antibodies to HSP60, HSP65, and HSP70. 

HSPs can serve as biomarkers for atrial fibrillation and can be a potential target to regularize proteostasis and decrease the substrate for atrial fibrillation [105]. HSP inducers/activators (GGA, L-glutamine) can be a promising therapy to prevent or reduce clinical atrial fibrillation, including post-operative atrial fibrillation. Several other drugs can prime HSPs, but their potential benefits, like cardioprotection, must be investigated in future human studies [106].

An animal study by Toga et al. showed a benefit from using Trandopril in left ventricular dysfunction after acute myocardial infarction by preventing a reduction in mitochondrial function, reducing reactive oxygen stress, and altering the production of HSP60 [107].

There are very few studies comparing HSPs with classical biomarkers such as NT-proBNP or high-sensitivity troponins. A small study showed that HSP27 is a good predictor of chronic heart failure prognosis, independent of NT-proBNP levels, left ventricular ejection fraction, smoking, or renal function [108]. As shown in a study by Li Z et al. [60], HSP70 may be an early marker of cardiac remodeling and aid in the early diagnosis of heart failure. This may facilitate the early introduction of treatments to reduce symptoms or reverse heart disease. 

## 16. Conclusions

Under normal conditions, intracellular HSPs act as molecular chaperones that fold, assemble, localize, secrete, and translocate cellular proteins. Various stress mechanisms, such as mechanical stress, environmental stress (heat, cold, UV light), and infections, markedly induce their expression. The detection of extracellular HSPs is an emerging field of study. Although there were initial controversies regarding their role, it is now recognized that these molecules are not artifacts and reflect novel biological phenomena.

The primary role of Ec-HSPs appears to be at the level of signaling or cellular communication. Intracellular HSPs primarily have chaperone activity. In common cardiac pathologies such as cardiac hypertrophy, heart failure, and reperfusion injury, an elevation of Ec-HSPs is noted. The evidence for the role of extracellular Hsp70, Hsp90, and BAG-3 in the pathogenesis of heart failure and other chronic cardiac disorders is naïve at best, with some conflicting results; however, a cardioprotective effect has been observed. The existing gaps in knowledge about this fascinating biological phenomenon need further research.

## Figures and Tables

**Table 1:** Classification of HSPs.

Family	Important Members
Hsp100	Hsp105 (HSPH1) Hsp110 (HSPH2) Grp170 (HSPH4)
Hsp90	Hsp90α (HSPC2) Hsp90β (HSPC3) Grp94 (HSPC4)
Hsp70	Hsp70 (Hsp72) (HSPA1) Hsc70 (Hsp73) (HSPA8) Grp78 (BIP) (HSPA5) Utp (Grp75) (HSPA9)
Hsp40	Hsp40 (Dnaj) DNAJB1
	αCrystallin (HSPB4)
	Hsp25 (HSPB1)
Small Hsp	Hsp27 (HSPB2)
	Hsp20 (HSPB6)
	Hsp22 (HSPB8)
Chaperonins	GroEL (Hsp60) (HSPD1) GroES (HSPE1)

## Data Availability

Not applicable.
